# Cure of a Case of Open-Joint Where Fluid Had Escaped

**Published:** 1900-02

**Authors:** W. T. Campbell

**Affiliations:** Cincinnati, Ohio


### CURE OF A CASE OF OPEN-JOINT WHERE FLUID HAD ESCAPED.

By W. T. Campbell, D.V.S.,
CINCINNATI, OHIO.

On November 1st I was called to see a gray mare, which with her mate had run away and collided with an electric car. She was lying down on the street when I saw her, and I at once ordered her hauled to the stable in an ambulance. On examination I found, among other injuries, that she had an ugly lacerated wound on the left knee, penetrating the joint, from which the fluid had escaped. I at once cleaned the wound out with a solution of bichloride of mercury, and made it as aseptic as possible by removing all ragged edges. Following this I drew the edges of the capsule together with a fine needle and thread and covered this over with a coating of aristol and collodion. I then sprinkled the edges of the wound with aristol (powdered) and drew them together and applied another, but heavier, coating of aristol and collodion.

I ordered her kept quiet and applied a splint and a long bandage. After she had quieted down we placed her in a padded sling. On every third day we removed the splint and bandage and gave another coating. We kept her in the sling until she entirely recovered, and on January 1st I found her as well as ever, and she has not shown any lameness since, although she is working in a heavy coal-wagon.

